# Evaluation of the performance and gene expression of two strains of Japanese quail following supplementation with frankincense and *Melissa officinalis*

**DOI:** 10.1038/s41598-026-54797-2

**Published:** 2026-06-05

**Authors:** Ebtsam E. Elkhoriby, Hanaa M. Ghanem, Mohammed M. Fouda, Samer S. Ibrahim, Ahmed Ateya, Ayman E. Tahoon, Hend A. Radwan

**Affiliations:** 1 Department of Animal Wealth Development, Faculty of Veterinary Medicine, Mansoura University, Mansoura, Egypt; 2 Department of Behaviour and Animal Management, Faculty of Veterinary Medicine, Mansoura University, Mansoura, Egypt; 3 Department of Animal Husbandry and Wealth Development, Faculty of Veterinary Medicine, Badr University in Cairo (BUC), Badr City, Cairo 11829 Egypt

**Keywords:** Frankincense, *Melissa officinalis*, Japanese quail strains, Performance, Economic efficiency, Gene expression, Biotechnology, Genetics, Molecular biology

## Abstract

This study evaluated the effects of dietary supplementation with frankincense (FR) and *Melissa officinalis* (MO) on growth performance, meat quality, economic efficiency, and the expression of growth-, immunity-, and antioxidant-related genes in two strains of Japanese quail. A total of 300 fourteen-day-old female quails (150 brown, 150 white) were assigned to five treatments per strain: a control, two FR levels (10 and 12 g/L drinking water) and two MO levels (2.5 and 3 mL/L drinking water), in a completely randomized design with a 2 × 5 factorial arrangement for 28 days. Results revealed a significant treatment × strain interaction, indicating a strain-dependent response. In the brown strain, 10 and 12 g/L FR produced the most favorable responses, whereas in the white strain, 10 g/L FR and 3 mL/L MO were the most effective, resulting in improved growth performance and economic returns. At the treatment level, 10 g/L FR and 3 mL/L MO enhanced body weight, feed efficiency, dressing percentage, and economic indicators. These improvements were associated with upregulation of *IGF-1*, *GPX1*, and *IL-6*, along with downregulation of *MSTN*. At the strain level, the white strain outperformed the brown strain in productive traits and showed higher *IGF-1* expression with lower *MSTN* levels, whereas the brown strain exhibited relatively stronger *GPX1* and *IL-6* expressions. Therefore, these findings indicate that the effectiveness of phytogenic feed additives is strongly influenced by genetic background, highlighting the importance of optimizing both treatment level and strain selection to maximize productive performance, meat quality, and economic efficiency in Japanese quail. Specifically, FR at 10–12 g/L is recommended for brown quails, whereas 10 g/L FR or 3 mL/L MO is more suitable for white quails.

## Introduction

The increasing global demand for animal-based protein has intensified the search for efficient and sustainable sources of meat. Among these, Japanese quail (*Coturnix coturnix japonica*) has emerged as a promising candidate due to its rapid growth rate, low feed intake, early sexual maturity, and minimal housing requirements, offering several advantages over conventional poultry species^1–3^.

Traditionally, antibiotic growth promoters (AGPs) have been used in poultry production to improve growth performance, feed efficiency, and gut health by modulating intestinal microbiota^4^. However, growing concerns regarding antibiotic residues, noncompliance with withdrawal periods, and their potential health risks, including allergic reactions, teratogenicity, carcinogenicity, and most notably, the emergence of antimicrobial resistance have prompted a global shift toward safer alternatives^5,6^. In response, phytogenic feed additives (PFAs) natural bioactive compounds derived from herbs, spices, and plants have gained increasing attention as potential substitutes for AGPs. These compounds are known for their antioxidant, antimicrobial, anti-inflammatory, and growth-promoting properties and are widely accepted in organic and sustainable animal production systems^7^.

Two promising PFAs include frankincense (*Boswellia* spp.) and *Melissa officinalis* (lemon balm). Frankincense, a resin obtained from Boswellia trees, has demonstrated multiple biological effects in poultry, including improved growth performance, feed utilization, meat quality, immune modulation, and safety with no detectable residues^8,9^. Similarly, *Melissa officinalis*, a medicinal herb of the *Lamiaceae* family, is rich in polyphenols and flavonoids and is known for its antioxidant, anti-inflammatory, antimicrobial, and immunomodulatory activities^10^. It has been proposed as a natural growth enhancer and meat quality improver, even in organic poultry farming^11,12^.

Moreover, the evaluation of certain genes involved in physiological regulation can provide insights into the mechanisms underlying the effects of PFAs. The genes *IGF-1* (*Insulin-like Growth Factor 1*), *MSTN* (*Myostatin*), *IL-6* (*Interleukin-6*), and *GPX1* (*Glutathione Peroxidase 1*) play essential roles in regulating key physiological processes in poultry. *IGF-1* is a major growth-related gene involved in cell proliferation and muscle development, whereas *MSTN* acts as a negative regulator of muscle growth. *IL-6* is an important cytokine involved in immune regulation and inflammatory responses, while *GPX1* is a key antioxidant enzyme that protects cells against oxidative stress^13–16^.

Despite the growing interest in PFAs as natural alternatives to antibiotic growth promoters in poultry nutrition, most previous studies have primarily focused on their effects on growth performance and basic production traits. Limited information is available regarding the combined effects of frankincense and *Melissa officinalis* supplementation on productive performance, meat quality, economic efficiency, and molecular responses in Japanese quail. Moreover, little attention has been given to evaluating the differential responses of different quail strains to phytogenic supplementation at the gene expression level. Investigating the expression of key genes involved in growth (*IGF-1*,* MSTN*), antioxidant defense (*GPX1*), and immune response (*IL-6*) may provide valuable insights into the biological mechanisms underlying the effects of these herbal additives.

Therefore, the objective of the present experiment was to evaluate the effects of drinking water supplementation with frankincense or *Melissa officinalis* at different concentrations on growth performance, carcass traits, meat quality, economic efficiency, and the expression of *IGF-1*, *MSTN*, *GPX1*, and *IL-6* genes in brown and white Japanese quail strains.

## Results

### Growth performance

Considering the factorial experimental design (2 × 5), the results are presented by prioritizing the interaction between treatment and strain (T × S), followed by the main effects of treatment (T) and strain (S).

A significant T × S interaction was observed for BW at days 28, 35, and 42 (*P* < 0.05; Table 1). At day 42, the response to dietary treatments differed between strains. In the brown strain, the greatest BW was observed in birds treated with 12 g FR (255.35 g) and 10 g FR (250.78 g), whereas in the white strain, the highest BW values were recorded in birds receiving 10 g FR (265.95 g) and 3 ml MO (253.79 g). Regarding the main effect of treatment, no significant differences were observed among the treatment groups at 14 and 21 days of age. However, from day 28 onwards, dietary interventions had a significant effect on BW (*P* = 0.008 at day 28; *P* < 0.001 at days 35 and 42). At day 42, birds receiving 10 g FR, 12 g FR, and 3 ml MO showed significantly higher BW (258.36 g, 246.84 g, and 243.17 g, respectively) compared to the control group (235.48 g). For the strain effect, the white strain had significantly higher final BW than the brown strain (246.97 g vs. 240.14 g, *P* < 0.001).

**Table 1:** Effect of different levels of experimental treatments of PFAs (Frankincense, *Melissa officinalis*) on body weights of two different commercial Japanese quail strains.

Body weights (g)						
Least squares means ± Standard error						
Age (day)		BW14	BW21	BW28	BW35	BW42
Treatments-strain interaction (T*S)						
Brown	Control	57.44 ± 0.06	116.84 ± 0.25	168.53 ± 1.47^b^	206.78 ± 0.73^b^	232.66 ± 0.85^b^
	10 g FR	57.42 ± 0.06	115.44 ± 0.11	178.36 ± 2.32^a^	222.22 ± 2.80^a^	250.78 ± 2.89^a^
	12 g FR	57.48 ± 0.04	117.01 ± 1.70	180.23 ± 2.45^a^	226.01 ± 0.53^a^	255.35 ± 3.12^a^
	2.5 ml MO	57.42 ± 0.05	116.20 ± 1.33	166.34 ± 2.19^b^	206.33 ± 1.29^b^	229.37 ± 2.09^b^
	3 ml MO	57.46 ± 0.06	118.39 ± 0.43	168.61 ± 0.53^b^	206.67 ± 0.76^b^	232.56 ± 1.16^b^
White	Control	57.51 ± 0.04	116.99 ± 0.16	175.35 ± 1.33^ns^	211.01 ± 2.03^b^	238.29 ± 2.65^c^
	10 g FR	57.48 ± 0.07	118.17 ± 1.98	180.78 ± 3.23^ns^	225.89 ± 3.51^a^	265.95 ± 3.64^a^
	12 g FR	57.33 ± 0.12	118.75 ± 3.03	175.38 ± 1.77^ns^	210.94 ± 1.94^b^	238.32 ± 1.11^c^
	2.5 ml MO	57.50 ± 0.08	118.75 ± 2.29	175.54 ± 0.65^ns^	211.11 ± 2.41^b^	238.47 ± 1.05^c^
	3 ml MO	57.41 ± 0.12	118.41 ± 1.76	179.19 ± 4.94^ns^	221.63 ± 2.06^ab^	253.79 ± 2.71^b^
Treatment effect (T)						
Control		57.47 ± 0.03	116.91 ± 0.14	171.94 ± 1.76^b^	208.90 ± 1.35^c^	235.48 ± 1.77^c^
10 g FR		57.45 ± 0.04	116.81 ± 1.08	179.57 ± 1.86^a^	224.06 ± 2.17^a^	258.36 ± 3.98^a^
12 g FR		57.41 ± 0.07	117.88 ± 1.60	177.80 ± 1.73^ab^	218.48 ± 3.49^ab^	246.84 ± 4.09^b^
2.5 ml MO		57.46 ± 0.05	117.47 ± 1.32	170.94 ± 2.30^b^	208.72 ± 1.62^c^	233.92 ± 2.29^c^
3 ml MO		57.43 ± 0.06	118.40 ± 0.81	173.90 ± 3.25^ab^	214.15 ± 3.49^bc^	243.17 ± 4.93^b^
Strain effect (S)						
Brown		57.44 ± 0.02	116.78 ± 0.46	172.41 ± 1.69^b^	213.60 ± 2.38	240.14 ± 2.98^b^
White		57.44 ± 0.04	118.21 ± 0.80	177.25 ± 1.23^a^	216.12 ± 1.94	246.97 ± 3.14^a^
P-value						
T*S		0.510	0.864	0.031	< 0.001	< 0.001
T		0.916	0.850	0.008	< 0.001	< 0.001
S		0.998	0.177	0.005	0.064	< 0.001

^abc^Means within the same column and for the same effect with different superscripts are significantly different (*P* ≤ 0.05).

FR, Frankincense; MO, *Melissa officinalis*; T, Treatment effect; S, Strain effect; TS, Treatments-Strain interaction.

A significant T × S interaction was also observed for BWG during the later growth stages and over the entire experimental period (*P* < 0.001; Table 2). In the brown strain, the highest BWG was observed with 12 g FR (197.87 g) and 10 g FR (193.36 g), whereas in the white strain, the greatest BWG was recorded with 10 g FR (208.47 g) and 3 ml MO (196.38 g). The treatment effect became significant from the third week onwards, where birds receiving 10 g FR consistently exhibited the highest BWG during the later growth phases, with a total BWG of 200.91 g, compared to the control (178.00 g) and 2.5 ml MO (176.46 g) groups. The 12 g FR and 3 ml MO groups also showed notable improvements. The strain effect was significant, with the white strain outperforming the brown strain during both the final week and the overall period (*P* < 0.001).

**Table 2:** Effect of different levels of experimental treatments of PFAs (Frankincense, *Melissa officinalis*) on body weight gain of two different commercial Japanese quail strains.

Body weight gain (g)						
Least squares means ± Standard error						
		2–3 weeks	3–4 weeks	4–5 weeks	5–6 weeks	2–6 weeks
Treatments-strain interaction (T*S)						
Brown	Control	59.40 ± 0.31	51.69 ± 1.44^b^	38.26 ± 1.76^b^	25.87 ± 1.38^ns^	175.22 ± 0.88^b^
	10 g FR	58.02 ± 0.06	62.91 ± 2.42^a^	43.87 ± 0.88^ab^	28.56 ± 0.40^ns^	193.36 ± 2.94^a^
	12 g FR	59.52 ± 1.74	63.22 ± 0.87^a^	45.78 ± 1.93^a^	29.34 ± 2.76^ns^	197.87 ± 3.14^a^
	2.5 ml MO	58.78 ± 1.35	50.14 ± 2.42^b^	39.99 ± 1.54^ab^	23.03 ± 2.17^ns^	171.94 ± 2.04^b^
	3 ml MO	60.93 ± 0.39	50.22 ± 0.91^b^	38.06 ± 1.09^b^	25.89 ± 0.40^ns^	175.10 ± 1.14^b^
White	Control	59.48 ± 0.14	58.36 ± 1.18^ns^	35.66 ± 0.98^ns^	27.29 ± 1.20^b^	180.79 ± 2.65^c^
	10 g FR	60.69 ± 1.94	62.61 ± 5.19^ns^	45.11 ± 0.91^ns^	40.06 ± 0.85^a^	208.47 ± 3.68^a^
	12 g FR	61.42 ± 2.99	56.63 ± 2.27^ns^	35.57 ± 1.58^ns^	27.38 ± 1.00^b^	180.99 ± 1.13^c^
	2.5 ml MO	61.25 ± 2.21	56.79 ± 1.83^ns^	35.57 ± 2.68^ns^	27.36 ± 1.82^b^	180.97 ± 0.97^c^
	3 ml MO	61.00 ± 1.88	60.78 ± 6.57^ns^	42.43 ± 6.32^ns^	32.16 ± 1.27^b^	196.38 ± 2.80^b^
Treatment effect (T)						
Control		59.44 ± 0.15	55.03 ± 1.71^ab^	36.96 ± 1.07^b^	26.58 ± 0.88^b^	178.00 ± 1.76^c^
10 g FR		59.36 ± 1.06	62.76 ± 2.56^a^	44.49 ± 0.63^a^	34.31 ± 2.61^a^	200.91 ± 3.98^a^
12 g FR		60.47 ± 1.61	59.93 ± 1.83^ab^	40.67 ± 2.54^ab^	28.36 ± 1.39^b^	189.43 ± 4.06^b^
2.5 ml MO		60.01 ± 1.28	53.47 ± 2.01^b^	37.78 ± 1.70^ab^	25.20 ± 1.59^b^	176.46 ± 2.26^c^
3 ml MO		60.97 ± 0.86	55.50 ± 3.79^ab^	40.25 ± 3.03^ab^	29.03 ± 1.52^b^	185.74 ± 4.95^b^
Strain effect (S)						
Brown		59.33 ± 0.46	55.64 ± 1.76	41.19 ± 1.00	26.54 ± 0.88^b^	182.70 ± 2.98^b^
White		60.77 ± 0.80	59.04 ± 1.63	38.87 ± 1.63	30.85 ± 1.41^a^	189.52 ± 3.14^a^

^abc^Means within the same column and for the same effect with different superscripts are significantly different (*P* ≤ 0.05).

FR, Frankincense; MO, *Melissa officinalis*; T, Treatment effect; S, Strain effect; TS, Treatments-Strain interaction.

Regarding FI, no significant T × S interaction was observed (*P* > 0.05; Table 3), and no significant differences were detected among treatment groups. However, the strain effect was significant, with white quails consuming more feed than brown quails during the 4–5 and 5–6-week intervals, as well as over the entire experimental period (*P* < 0.001).

**Table 3:** Effect of different levels of experimental treatments of PFAs (Frankincense, *Melissa officinalis*) on feed intake of two different commercial Japanese quail strains.

FI (g)						
Least squares means ± Standard error						
		2–3 weeks	3–4 weeks	4–5 weeks	5–6 weeks	2–6 weeks
Treatments-strain interaction (T*S)						
Brown	Control	115.17 ± 0.88	134.00 ± 2.29	155.33 ± 2.09	192.83 ± 1.42	597.33 ± 2.05
	10 g FR	116.83 ± 0.93	135.50 ± 2.29	151.83 ± 1.17	193.67 ± 2.95	597.83 ± 3.37
	12 g FR	117.67 ± 0.17	133.33 ± 1.42	152.00 ± 1.32	190.67 ± 0.67	593.67 ± 0.60
	2.5 ml MO	114.50 ± 1.89	134.00 ± 2.29	151.67 ± 1.01	193.67 ± 2.95	593.83 ± 4.76
	3 ml MO	117.67 ± 0.17	134.17 ± 1.92	152.83 ± 1.42	190.67 ± 0.67	595.33 ± 1.92
White	Control	117.67 ± 0.60	137.00 ± 0.50	157.33 ± 1.59	196.17 ± 1.30	608.17 ± 0.88
	10 g FR	116.33 ± 0.83	134.50 ± 1.44	156.33 ± 1.96	195.67 ± 1.48	602.83 ± 2.83
	12 g FR	116.33 ± 1.01	132.50 ± 1.80	154.17 ± 0.88	196.00 ± 1.44	599.00 ± 1.61
	2.5 ml MO	117.50 ± 0.00	135.50 ± 2.25	157.83 ± 0.73	196.50 ± 1.15	607.33 ± 1.92
	3 ml MO	116.77 ± 0.50	136.83 ± 2.42	153.00 ± 1.80	198.50 ± 0.29	605.10 ± 3.34
Treatment effect (T)						
Control		116.42 ± 0.74	135.50 ± 1.24	156.33 ± 1.26	194.50 ± 1.14	602.75 ± 2.62
10 g FR		116.58 ± 0.57	135.00 ± 1.23	154.08 ± 1.43	194.67 ± 1.54	600.33 ± 2.26
12 g FR		117.00 ± 0.55	132.92 ± 1.04	153.08 ± 0.86	193.33 ± 1.39	596.33 ± 1.42
2.5 ml MO		116.00 ± 1.08	134.75 ± 1.48	154.75 ± 1.49	195.08 ± 1.55	600.58 ± 3.79
3 ml MO		117.22 ± 0.31	135.50 ± 1.51	152.92 ± 1.03	194.58 ± 1.78	600.22 ± 2.78
Strain effect (S)						
Brown		116.37 ± 0.52	134.20 ± 0.81	152.73 ± 0.66^b^	192.30 ± 0.84^b^	595.60 ± 1.19^b^
White		116.92 ± 0.30	135.27 ± 0.82	155.73 ± 0.75^a^	196.57 ± 0.53^a^	604.49 ± 1.24^a^
P-value						
T*S		0.059	0.755	0.311	0.436	0.442
T		0.662	0.668	0.167	0.868	0.221
S		0.328	0.397	0.004	0.001	< 0.001

^abc^Means within the same column and for the same effect with different superscripts are significantly different (*P* ≤ 0.05).

FR, Frankincense; MO, *Melissa officinalis*; T, Treatment effect; S, Strain effect; TS, Treatments-Strain interaction.

A significant T × S interaction was observed for cumulative FCR (*P* < 0.001; Table 4). In the brown strain, the lowest FCR values were recorded with 12 g FR (3.00) and 10 g FR (3.09), whereas in the white strain, the best FCR values were observed with 10 g FR (2.89) and 3 ml MO (3.08). Significant treatment effects were detected from the third week onwards. Birds receiving 10 g FR consistently showed the most efficient feed utilization, recording the lowest total FCR (2.99), which was significantly better than the control (3.39) and 2.5 ml MO (3.41). The 12 g FR and 3 ml MO groups also demonstrated improved FCR values (3.16 and 3.24, respectively). The strain effect was also significant, with the white strain showing better overall feed efficiency compared to the brown strain (3.20 vs. 3.27; *P* = 0.026).

**Table 4:** Effect of different levels of experimental treatments of PFAs (Frankincense, *Melissa officinalis*) on FCR of two different commercial Japanese quail strains.

FCR						
Least squares means ± Standard error						
		2–3 weeks	3–4 weeks	4–5 weeks	5–6 weeks	2–6 weeks
Treatments-strain interaction (T*S)						
Brown	Control	1.94 ± 0.02	2.60 ± 0.11^ab^	4.08 ± 0.21^a^	7.49 ± 0.36^ns^	3.41 ± 0.02^a^
	10 g FR	2.01 ± 0.02	2.16 ± 0.12^b^	3.46 ± 0.04^ab^	6.79 ± 0.16^ns^	3.09 ± 0.06^b^
	12 g FR	1.98 ± 0.06	2.11 ± 0.05^bc^	3.34 ± 0.18^b^	6.63 ± 0.71^ns^	3.00 ± 0.05^b^
	2.5 ml MO	1.95 ± 0.08	2.69 ± 0.18^a^	3.80 ± 0.16^ab^	8.56 ± 0.80^ns^	3.46 ± 0.07^a^
	3 ml MO	1.93 ± 0.01	2.67 ± 0.06^a^	4.02 ± 0.09^ab^	7.37 ± 0.12^ns^	3.40 ± 0.03^a^
White	Control	1.98 ± 0.01	2.35 ± 0.05^ns^	4.42 ± 0.12 ^ns^	7.22 ± 0.34^a^	3.37 ± 0.05^a^
	10 g FR	1.92 ± 0.07	2.18 ± 0.18^ns^	3.47 ± 0.06^ns^	4.89 ± 0.13^b^	2.89 ± 0.05^b^
	12 g FR	1.90 ± 0.08	2.35 ± 0.07^ns^	4.35 ± 0.18^ns^	7.18 ± 0.29^a^	3.31 ± 0.02^a^
	2.5 ml MO	1.92 ± 0.07	2.39 ± 0.11^ns^	4.49 ± 0.34^ns^	7.25 ± 0.50^a^	3.36 ± 0.01^a^
	3 ml MO	1.92 ± 0.06	2.30 ± 0.23^ns^	3.78 ± 0.58^ns^	6.19 ± 0.20^ab^	3.08 ± 0.06^b^
Treatment effect (T)						
Control		1.96 ± 0.01	2.47 ± 0.08^a^	4.25 ± 0.13^a^	7.35 ± 0.23^a^	3.39 ± 0.03^a^
10 g FR		1.97 ± 0.04	2.17 ± 0.09^b^	3.47 ± 0.03^b^	5.84 ± 0.43^b^	2.99 ± 0.06^c^
12 g FR		1.94 ± 0.05	2.23 ± 0.06^ab^	3.84 ± 0.25^ab^	6.91 ± 0.36^ab^	3.16 ± 0.07^b^
2.5 ml MO		1.94 ± 0.05	2.54 ± 0.12^a^	4.15 ± 0.23^ab^	7.90 ± 0.51^a^	3.41 ± 0.04^a^
3 ml MO		1.92 ± 0.03	2.49 ± 0.14^a^	3.90 ± 0.27^ab^	6.78 ± 0.29^ab^	3.24 ± 0.08^b^
Strain Effect (S)						
Brown		1.96 ± 0.02	2.45 ± 0.08^ab^	3.74 ± 0.10^b^	7.37 ± 0.27^a^	3.27 ± 0.05^a^
White		1.93 ± 0.02	2.31 ± 0.06^b^	4.10 ± 0.16^a^	6.55 ± 0.27^b^	3.20 ± 0.05^b^
P-value						
T*S		0.755	0.154	0.118	0.073	< 0.001
T		0.938	0.029	0.041	0.002	< 0.001
S		0.326	0.120	0.032	0.007	0.026

^abc^Means within the same column and for the same effect with different superscripts are significantly different (*P* ≤ 0.05).

FR, Frankincense; MO, *Melissa officinalis*; T, Treatment effect; S, Strain effect; TS, Treatments-Strain interaction.

### Carcass traits

A significant T × S interaction was observed for dressing percentage, liver, and gizzard percentages (*P* < 0.05; Table 5), indicating a strain-dependent response. In the brown strain, no significant differences were observed in dressing percentage among treatments. However, birds supplemented with 10 g FR and 12 g FR showed higher liver percentages, while the highest gizzard percentage was recorded in the 2.5 ml MO group (2.92%). In contrast, the white strain exhibited the highest percentage in birds receiving 10 g FR (75.22%) and 3 ml MO (75.03%), whereas 12 g FR resulted in the highest liver percentage (3.09%). The greatest gizzard percentage was recorded in birds supplemented with 12 g FR (2.95%) and 2.5 ml MO (2.93%).

**Table 5:** Effect of different levels of experimental treatments of PFAs (Frankincense, *Melissa officinalis*) on carcass traits of two different commercial Japanese quail strains.

Carcass traits						
Least squares means ± Standard error						
Parameters%		Dressing	Liver	Gizzard	Heart	Spleen
Treatments-strain interaction (T*S)						
Brown	Control	72.58 ± 0.07^ns^	2.86 ± 0.01^c^	2.73 ± 0.02^b^	0.94 ± 0.03	0.11 ± 0.00^ab^
	10 g FR	74.00 ± 0.65^ns^	3.34 ± 0.04^a^	2.71 ± 0.00^b^	0.98 ± 0.03	0.10 ± 0.01^b^
	12 g FR	74.04 ± 0.48^ns^	3.42 ± 0.03^a^	2.63 ± 0.06^b^	0.94 ± 0.06	0.10 ± 0.01^b^
	2.5 ml MO	72.71 ± 0.13^ns^	3.00 ± 0.00^b^	2.92 ± 0.05^a^	1.00 ± 0.07	0.13 ± 0.00^a^
	3 ml MO	72.74 ± 0.05^ns^	3.10 ± 0.01 ^b^	2.78 ± 0.04^ab^	1.05 ± 0.05	0.14 ± 0.00^a^
White	Control	73.51 ± 0.46^b^	2.79 ± 0.01^e^	2.76 ± 0.01^b^	1.00 ± 0.07	0.10 ± 0.01^b^
	10 g FR	75.22 ± 0.09^a^	2.87 ± 0.01^d^	2.69 ± 0.01^c^	1.01 ± 0.05	0.10 ± 0.01^b^
	12 g FR	73.59 ± 0.06^b^	3.09 ± 0.01^a^	2.95 ± 0.01^a^	1.04 ± 0.05	0.11 ± 0.00^ab^
	2.5 ml MO	72.74 ± 0.07^b^	2.94 ± 0.01^c^	2.93 ± 0.01^a^	0.94 ± 0.04	0.14 ± 0.01^a^
	3 ml MO	75.03 ± 0.43^a^	3.03 ± 0.02^b^	2.80 ± 0.01^b^	0.98 ± 0.13	0.13 ± 0.00^ab^
Treatment effect (T)						
Control		73.05 ± 0.29^bc^	2.82 ± 0.02^d^	2.74 ± 0.01^bc^	0.97 ± 0.04	0.11 ± 0.01^b^
10 g FR		74.61 ± 0.40^a^	3.11 ± 0.11^b^	2.70 ± 0.01^c^	1.00 ± 0.03	0.10 ± 0.00^b^
12 g FR		73.81 ± 0.24^ab^	3.26 ± 0.07^a^	2.79 ± 0.08^b^	0.99 ± 0.04	0.10 ± 0.00^b^
2.5 ml MO		72.73 ± 0.07^c^	2.97 ± 0.01^c^	2.92 ± 0.02^a^	0.97 ± 0.04	0.14 ± 0.01^a^
3 ml MO		73.89 ± 0.55^ab^	3.06 ± 0.02^b^	2.79 ± 0.02^b^	1.01 ± 0.07	0.14 ± 0.00^a^
Strain effect (S)						
Brown		73.21 ± 0.22^b^	3.14 ± 0.06^a^	2.75 ± 0.03^b^	0.98 ± 0.02	0.12 ± 0.00
White		74.02 ± 0.28^a^	2.94 ± 0.03^b^	2.83 ± 0.03^a^	1.00 ± 0.03	0.12 ± 0.01
P-value						
T*S		0.005	< 0.001	< 0.001	0.631	0.490
T		< 0.001	< 0.001	< 0.001	0.949	< 0.001
S		0.001	< 0.001	0.001	0.721	0.654

^abc^Means within the same column and for the same effect with different superscripts are significantly different (*P* ≤ 0.05).

FR, Frankincense; MO, *Melissa officinalis*; T, Treatment effect; S, Strain effect; TS, Treatments-Strain interaction.

At the treatment level, significant differences were detected for dressing, liver, gizzard, and spleen percentages (*P* < 0.001). The 10 g FR group achieved the highest dressing percentage (74.61%), while the control showed the lowest (73.05%). Liver’s percentage was highest in the 12 g FR group (3.26%) and lowest in the control (2.82%). The 2.5 ml MO treatment resulted in the greatest gizzard percentage (2.92%), whereas both 2.5 and 3 ml MO significantly increased spleen percentage (0.14%) compared to the control (0.11%). Between strains, the white quail exhibited significantly higher dressing (74.02%) and gizzard percentages (2.83%), whereas the brown strain showed a higher liver percentage (3.14%) (*P* < 0.05).

### Sensory meat quality evaluation

The results of the sensory evaluation of meat quality are shown in Table 6. No significant T × S interaction was detected for any of the sensory attributes (*P* > 0.05; Table 6), indicating a consistent response across both quail strains.

**Table 6:** Effect of different levels of experimental treatments of PFAs (Frankincense and *Melissa officinalis*) on sensory meat quality of two different strain of commercial Japanese quails.

Sensory meat quality							
Least squares means ± Standard error							
Parameters		Color	Aroma	Juiciness	Tenderness	Taste	Overall acceptability
Treatments-strain interaction (T*S)							
Brown	Control	7.03 ± 0.72	6.53 ± 0.23^b^	7.53 ± 0.09	7.03 ± 0.27^b^	7.82 ± 0.51	7.62 ± 0.13
	10 g FR	7.67 ± 0.18	8.32 ± 0.26^a^	8.10 ± 0.21	8.13 ± 0.47^ab^	8.02 ± 0.53	7.99 ± 0.50
	12 g FR	7.47 ± 0.24	8.03 ± 0.55^a^	7.97 ± 0.49	8.21 ± 0.38^ab^	7.91 ± 0.46	8.25 ± 0.25
	2.5 ml MO	8.13 ± 0.07	8.17 ± 0.09^a^	8.37 ± 0.09	8.43 ± 0.19^ab^	8.30 ± 0.00	8.23 ± 0.03
	3 ml MO	8.60 ± 0.06	8.63 ± 0.12^a^	8.63 ± 0.20	8.93 ± 0.07^a^	8.20 ± 0.20	8.50 ± 0.15
White	Control	7.07 ± 0.47^c^	7.50 ± 0.25^b^	7.37 ± 0.38	7.37 ± 0.23	7.40 ± 0.23^c^	7.40 ± 0.26
	10 g FR	7.27 ± 0.03^bc^	7.93 ± 0.35^ab^	8.46 ± 0.44	8.17 ± 0.49	7.97 ± 0.15^bc^	8.17 ± 0.19
	12 g FR	7.20 ± 0.12^c^	8.20 ± 0.29^ab^	8.16 ± 0.46	8.10 ± 0.38	8.17 ± 0.19^ab^	8.06 ± 0.46
	2.5 ml MO	8.27 ± 0.13^ab^	8.47 ± 0.15^ab^	8.23 ± 0.12	8.53 ± 0.29	8.27 ± 0.15^ab^	8.27 ± 0.34
	3 ml MO	8.37 ± 0.09^a^	8.67 ± 0.12^a^	8.77 ± 0.12	8.70 ± 0.15	8.80 ± 0.06^a^	8.47 ± 0.18
Treatment effect (T)							
Control		7.05 ± 0.38^c^	7.02 ± 0.27^b^	7.45 ± 0.18^b^	7.20 ± 0.18^b^	7.61 ± 0.27	7.51 ± 0.14^b^
10 g FR		7.47 ± 0.12^bc^	8.12 ± 0.21^a^	8.28 ± 0.23^ab^	8.15 ± 0.30^ab^	8.00 ± 0.25	8.08 ± 0.24^ab^
12 g FR		7.33 ± 0.13^bc^	8.12 ± 0.28^a^	8.07 ± 0.31^ab^	8.16 ± 0.24^ab^	8.04 ± 0.23	8.16 ± 0.24^ab^
2.5 ml MO		8.20 ± 0.07^ab^	8.32 ± 0.10^a^	8.30 ± 0.07^ab^	8.48 ± 0.16^a^	8.28 ± 0.07	8.25 ± 0.15^ab^
3 ml MO		8.48 ± 0.07^a^	8.65 ± 0.08^a^	8.70 ± 0.11^a^	8.82 ± 0.09^a^	8.50 ± 0.16	8.48 ± 0.10^a^
Strain effect (S)							
Brown		7.78 ± 0.20	7.94 ± 0.23	8.12 ± 0.14	8.15 ± 0.20	8.05 ± 0.16	8.12 ± 0.13
White		7.63 ± 0.17	8.15 ± 0.14	8.20 ± 0.18	8.17 ± 0.18	8.12 ± 0.14	8.07 ± 0.15
P-value							
T*S		0.885	0.210	0.891	0.920	0.547	0.951
T		< 0.001	< 0.001	0.009	0.001	0.084	0.036
S		0.441	0.229	0.687	0.904	0.728	0.808

^abc^Means within the same column and for the same effect with different superscripts are significantly different (*P* ≤ 0.05).

FR, Frankincense; MO, *Melissa officinalis*; T, Treatment effect; S, Strain effect; TS, Treatments-Strain interaction.

At the treatment level, dietary supplementation had a significant positive effect on most sensory traits, including color (*P* < 0.001), aroma (*P* < 0.001), juiciness (*P* = 0.009), tenderness (*P* = 0.001), and overall acceptability (*P* = 0.036), while taste was not significantly affected (*P* > 0.05). All additive treatments 10 g FR, 12 g FR, 2.5 ml MO, and 3 ml MO significantly enhanced aroma and tenderness scores compared to the control. Notably, the 12 g FR, 2.5 ml MO, and 3 ml MO treatments yielded significantly higher scores for overall acceptability. MO at both 2.5 ml and 3 ml concentrations significantly improved meat color. Regarding the strain effect, no significant differences were observed between brown and white quails for any of the sensory traits.

### Economic evaluation

Table 7 outlines the impact of dietary treatments on production costs and economic returns. A significant T × S interaction was detected for TR and NR (*P* < 0.001), indicating a strain-dependent economic response. In the brown strain, the highest TR was observed in birds receiving 10 g FR and 12 g FR, whereas no clear differences were detected in NR among treatments. In contrast, in the white strain, the highest TR and NR were achieved with 10 g FR, followed by 3 ml MO. At the treatment level, significant differences were observed for all economic parameters (*P* < 0.001). The 12 g FR group showed the highest TVC and TC, whereas the control group had the lowest costs. Birds receiving 10 g FR, 12 g FR, and 3 ml MO exhibited higher TR compared to the control. However, only the 10 g FR treatment resulted in a significantly higher NR, while 12 g FR showed a lower NR than the control. At the strain level, white quails exhibited significantly higher costs, returns, and net return compared to the brown strain (*P* < 0.001).

**Table 7:** Effect of different levels of experimental treatments of PFAs (Frankincense and *Melissa officinalis*) on production cost and economic return of two different commercial Japanese quail strains.

Economic parameters					
Least squares means ± standard error					
Parameters (EGP / quail)		TVC	TC	TR	NR
Treatments-strain interaction (T*S)					
Brown	Control	10.69 ± 0.01^d^	11.64 ± 0.01^d^	14.18 ± 0.05^b^	2.54 ± 0.05^ns^
	10 g FR	11.73 ± 0.03^b^	12.68 ± 0.03^b^	15.27 ± 0.17^a^	2.59 ± 0.20^ns^
	12 g FR	12.18 ± 0.01^a^	13.14 ± 0.01^a^	15.54 ± 0.19^a^	2.40 ± 0.19^ns^
	2.5 ml MO	10.89 ± 0.05^c^	11.84 ± 0.05^c^	13.98 ± 0.13^b^	2.14 ± 0.17^ns^
	3 ml MO	11.00 ± 0.02^c^	11.96 ± 0.02^c^	14.17 ± 0.07^b^	2.22 ± 0.09^ns^
White	Control	10.83 ± 0.01^d^	11.78 ± 0.01^d^	14.52 ± 0.16 ^c^	2.73 ± 0.16^ab^
	10 g FR	11.78 ± 0.03^b^	12.73 ± 0.03^b^	16.18 ± 0.22^a^	3.45 ± 0.21^a^
	12 g FR	12.19 ± 0.03^a^	13.14 ± 0.03^a^	14.52 ± 0.07^c^	1.38 ± 0.07^c^
	2.5 ml MO	11.00 ± 0.02^c^	11.96 ± 0.02^c^	14.53 ± 0.06^c^	2.57 ± 0.05^ab^
	3 ml MO	11.06 ± 0.05^c^	12.02 ± 0.05^c^	15.45 ± 0.16^b^	3.43 ± 0.21^a^
Treatment effect (T)					
Control		10.76 ± 0.03^e^	11.71 ± 0.03^e^	14.35 ± 0.11^c^	2.64 ± 0.09^ab^
10 g FR		11.75 ± 0.02^b^	12.71 ± 0.02^b^	15.72 ± 0.24^a^	3.02 ± 0.23^a^
12 g FR		12.18 ± 0.01^a^	13.14 ± 0.01^a^	15.03 ± 0.25^b^	1.89 ± 0.25^c^
2.5 ml MO		10.94 ± 0.03^d^	11.90 ± 0.03^d^	14.26 ± 0.14^c^	2.36 ± 0.12^b^
3 ml MO		11.03 ± 0.03^c^	11.99 ± 0.03^c^	14.81 ± 0.30^b^	2.82 ± 0.29^a^
Strain effect (S)					
Brown		11.30 ± 0.15^b^	12.25 ± 0.15^b^	14.63 ± 0.18^b^	2.38 ± 0.07^b^
White		11.37 ± 0.14^a^	12.33 ± 0.14^a^	15.04 ± 0.19^a^	2.71 ± 0.21^a^
P-value					
T*S		0.143	0.143	< 0.001	< 0.001
T		< 0.001	< 0.001	< 0.001	< 0.001
S		< 0.001	< 0.001	< 0.001	0.003

^abc^Means within the same column and for the same effect with different superscripts are significantly different (*P* ≤ 0.05).

FR, Frankincense; MO, *Melissa officinalis*; EGP: Egyptian pound; TVC, Total variable cost, TC, Total cost; TR, Total return; NR, Net return.

As shown in Table 8, a significant T × S interaction was observed for GM, RGM, and EE% (*P* < 0.001; Table 8). In the brown strain, economic values were generally moderate across treatments, with no clear superiority over control. In contrast, in the white strain, birds receiving 10 g FR and 3 ml MO achieved the highest economic values (GM: 4.40 and 4.38 EGP; RGM: 1.20 and 1.19; EE%: 49.03% and 54.30%, respectively), whereas 12 g FR resulted in the lowest values across all parameters. At the treatment level, birds fed 10 g FR and 3 ml MO showed the highest GM (3.97 and 3.78 EGP, respectively), both exceeding the control (3.59 EGP; *P* < 0.001). The lowest GM was recorded in the 12 g FR group (2.85 EGP). Similarly, RGM was highest in the 10 g FR (1.11) and 3 ml MO (1.05) groups, while the lowest value was observed with 12 g FR (0.80). For EE%, the highest value was recorded in the 3 ml MO group (44.88%), followed by the control (43.83%) and 10 g FR (43.05%), whereas the lowest EE% was observed in the 12 g FR group (25.42%). At the strain level, the white strain showed significantly higher GM and EE% compared to the brown strain (*P* = 0.003 and < 0.001, respectively), while no significant difference was detected in RGM between strains.

**Table 8:** Effect of different levels of dietary treatments of PFAs (Frankincense and *Melissa officinalis*) on economic efficiency of two different commercial Japanese quail strains.

Economic efficiency				
Least squares means ± Standard error				
Parameters (EGP / quail)		GM	RGM	EE%
Treatments-strain interaction (T*S)				
Brown	Control	3.49 ± 0.05^ns^	1.00 ± 0.00^ns^	42.71 ± 0.77^ns^
	10 g FR	3.54 ± 0.20^ns^	1.01 ± 0.06^ns^	37.08 ± 3.01^ns^
	12 g FR	3.36 ± 0.19^ns^	0.96 ± 0.06^ns^	32.30 ± 2.61^ns^
	2.5 ml MO	3.10 ± 0.17^ns^	0.89 ± 0.04^ns^	34.92 ± 2.91^ns^
	3 ml MO	3.17 ± 0.09^ns^	0.91 ± 0.04^ns^	35.47 ± 1.49^ns^
White	Control	3.69 ± 0.16^ab^	1.00 ± 0.00^a^	44.95 ± 2.71^ab^
	10 g FR	4.40 ± 0.21^a^	1.20 ± 0.10^a^	49.03 ± 2.97^ab^
	12 g FR	2.33 ± 0.07^c^	0.64 ± 0.04^b^	18.54 ± 1.04^c^
	2.5 ml MO	3.53 ± 0.05^b^	0.96 ± 0.05^a^	41.10 ± 0.70^b^
	3 ml MO	4.38 ± 0.21^a^	1.19 ± 0.09^a^	54.30 ± 3.68^a^
Treatment effect (T)				
Control		3.59 ± 0.09^ab^	1.00 ± 0.00^ab^	43.83 ± 1.35^a^
10 g FR		3.97 ± 0.23^a^	1.11 ± 0.07^a^	43.05 ± 3.27^a^
12 g FR		2.85 ± 0.25^c^	0.80 ± 0.08^c^	25.42 ± 3.32^b^
2.5 ml MO		3.31 ± 0.12^b^	0.92 ± 0.03^bc^	38.01 ± 1.92^a^
3 ml MO		3.78 ± 0.29^a^	1.05 ± 0.08^ab^	44.88 ± 4.57^a^
Strain effect (S)				
Brown		3.33 ± 0.07^b^	0.95 ± 0.02	36.50 ± 1.28^b^
White		3.67 ± 0.21^a^	1.00 ± 0.06	41.58 ± 3.43^a^
P-value				
T*S		< 0.001	< 0.001	< 0.001
T		< 0.001	< 0.001	< 0.001
S		0.003	0.236	< 0.001

^abc^Means within the same column and for the same effect with different superscripts are significantly different (*P* ≤ 0.05).

FR, Frankincense; MO, *Melissa officinalis*; EGP, Egyptian pound; GM, Gross margin; RGM, Relative gross margin; EE%, Economic efficiency %.

### Gene expression

#### Treatment × strain interaction

**Fig. 1:**
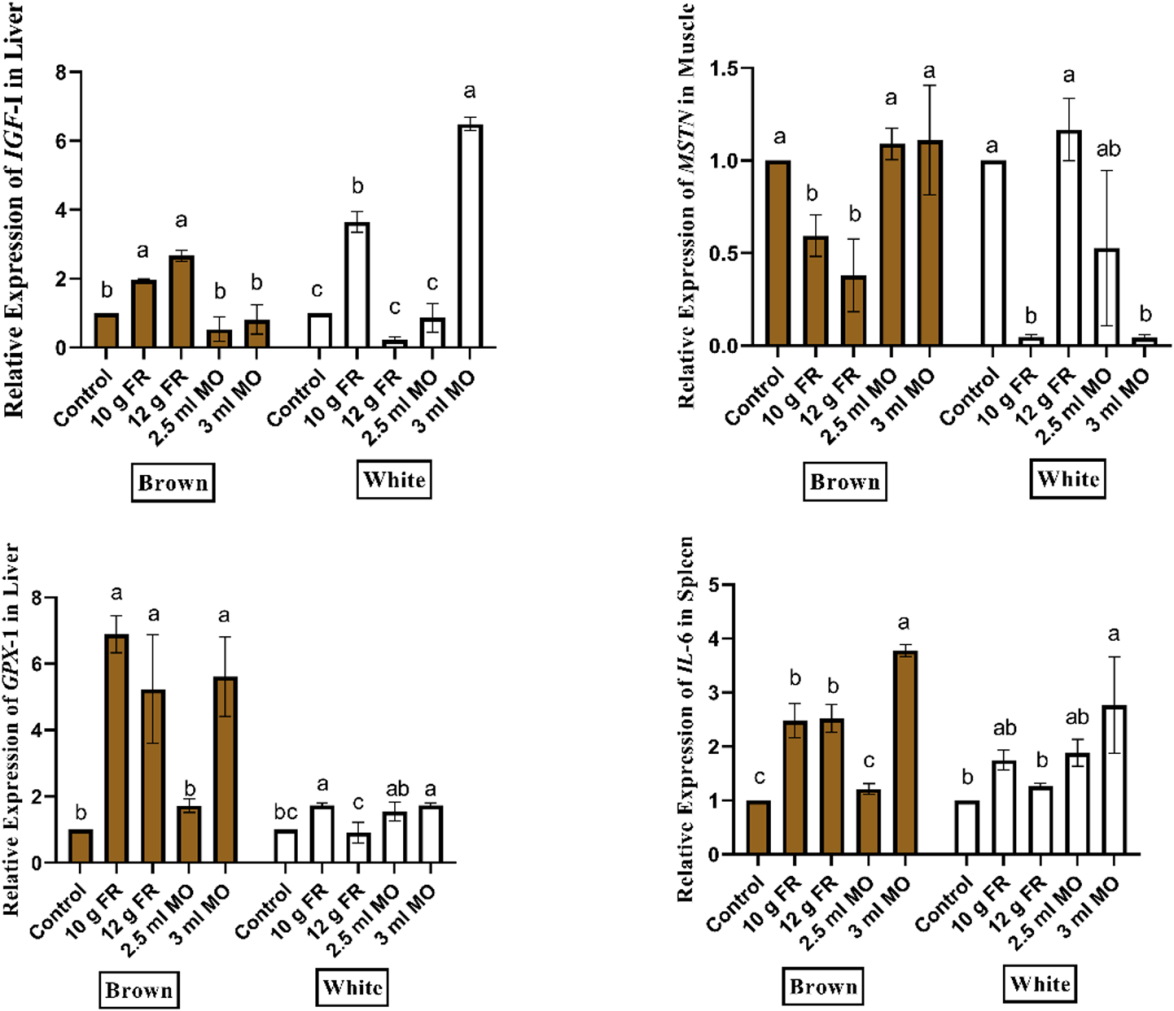
treatment × Strain interaction on growth (*IGF-I*, *MSTN*), antioxidant (*GPX1*), and immune (*IL-6*) gene expression in Japanese quail supplemented with phytogenic feed additives.

As indicated in Fig. 1, the interaction between T × S was significant (*P* < 0.05) for all genes studied. In the brown strain, the 10 and 12 g/L FR treatments produced the highest expression levels of *IGF-1*, *GPX1*, and *IL-6*, together with the lowest expression of *MSTN*. While, in the white strain, both the 3 mL/L MO and 10 g/L FR treatments resulted in marked upregulation of *IGF-1*, *GPX1*, and *IL-6*, accompanied by a strong downregulation of *MSTN*.

#### Treatment effect

**Fig. 2:**
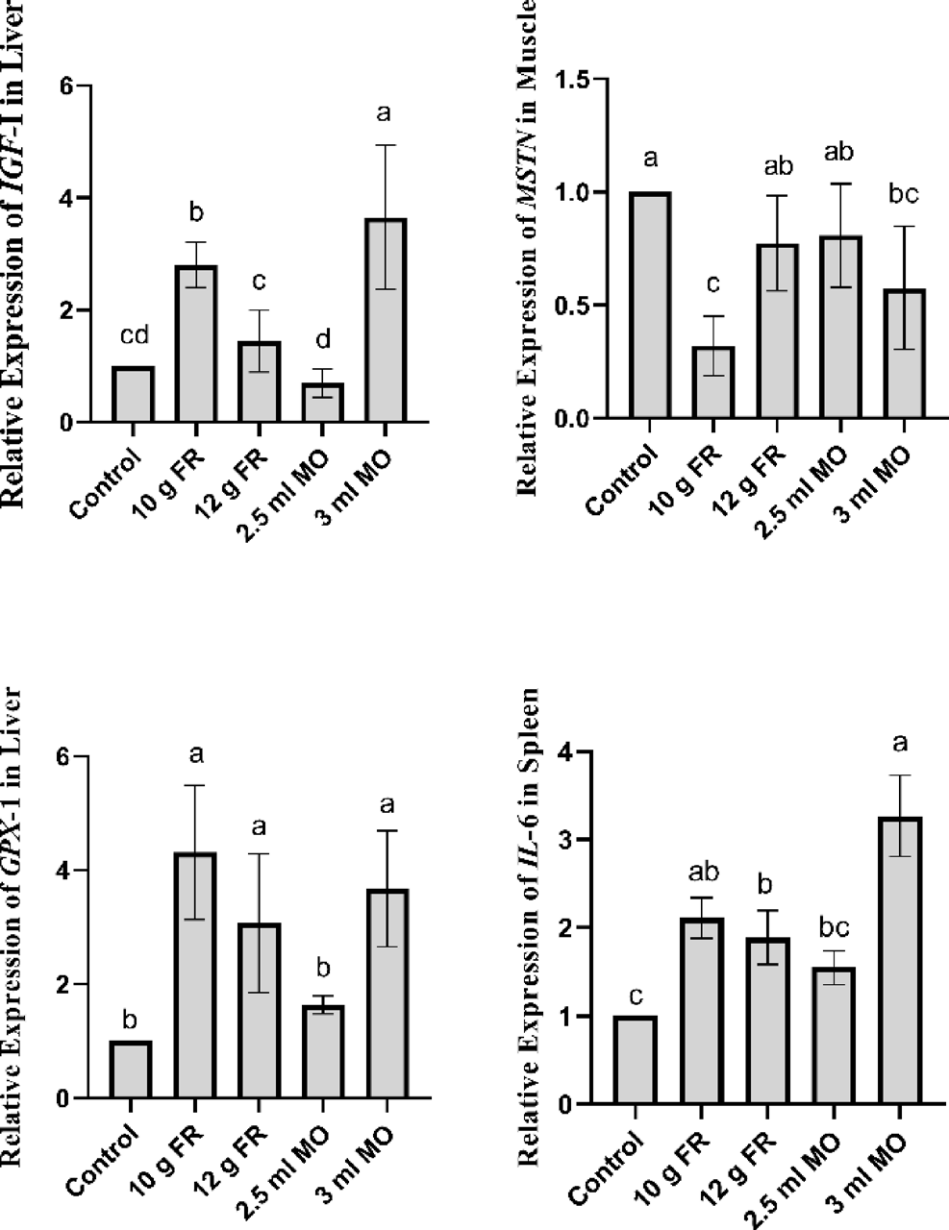
Treatment effects of frankincense and *Melissa officinalis* supplementation on growth (*IGF-I*, *MSTN*), antioxidant (*GPX1*), and immune (*IL-6*) gene expression in Japanese quail.

As illustrated in Fig. 2, Dietary supplementation with FR and MO significantly (*P* < 0.05) affected the expression of all studied genes (*IGF-1*, *MSTN*, *GPX1*, and *IL-6*). The 3 mL/L MO and 10 g/L FR treatments produced the most favorable responses, showing marked improvements in growth-related (*IGF-I* and low *MSTN*), antioxidant (*GPX1*), and immune-related (*IL-6*) gene expression followed by 12 g/L FR group compared with the control, and 2.5 mL/L MO.

#### Strain effect

**Fig. 3:**
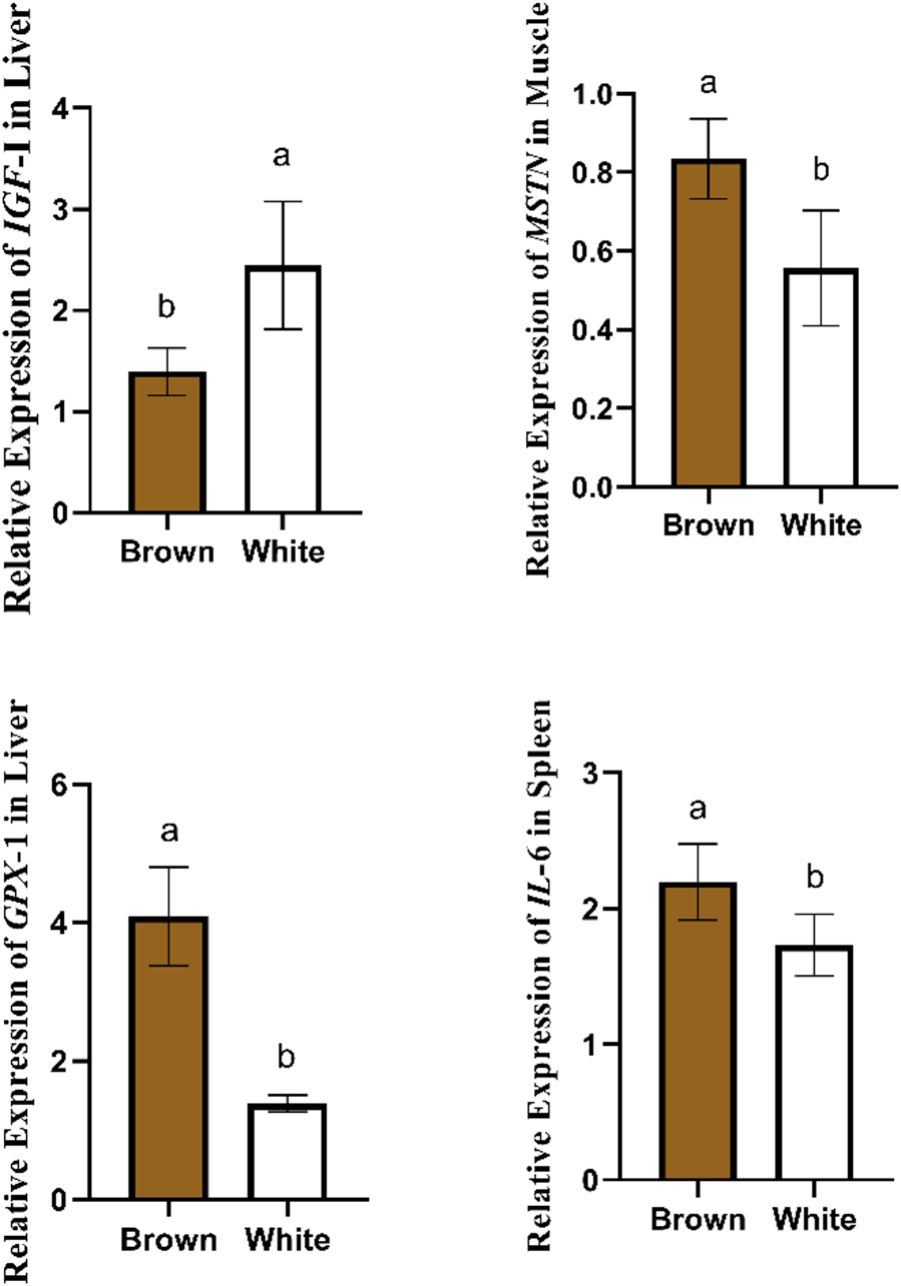
Strain-dependent differences in growth (*IGF-I*, *MSTN*), antioxidant (*GPX1*), and immune (*IL-6*) gene expression between brown and white Japanese quail supplemented with phytogenic feed additives.

As shown in Fig. 3, a significant (*P* < 0.05) strain difference was observed in gene expression.

The white quail strain showed higher expression of *IGF-1* and lower *MSTN* levels than the brown strain, indicating superior growth potential. Conversely, the brown strain expressed higher levels of *GPX1* and *IL-6*, reflecting stronger antioxidant and immune gene activity.

## Discussion

The T × S interaction revealed that white quails responded more favorably to 10 g FR and 3 ml MO, while brown quails showed a better response to 12 g FR, indicating strain-specific sensitivity to PFAs. These variations may be attributed to differences in genetic background, digestive physiology, and palatability preferences. For instance, the diminished performance of white quails on 12 g FR may relate to reduced palatability due to increased bitterness, while brown quails tolerated it better. These findings align with Kamel et al.^17^, who highlighted significant interactions for productivity traits with other PFAs. Significant differences in BW and BWG were not observed during the early phase (third week), likely due to a physiological adaptation period to the new environment and PFAs. However, by the fourth week onwards, treatment effects became prominent. Birds supplemented with 10 g FR, 12 g FR, and 3 ml MO showed consistently higher BW and BWG compared to the control. The positive impacts of FR align with a number of earlier research studies in broilers and rabbits, including Ismail et al.^18^, Al-Yasiry and Kiczorowska^19^ that documented notable increases in BW and BWG after FR supplementation. These improvements were ascribed to improved nutrient utilization and digestive efficiency mediated by boswellic acids. Similarly, Mohamed et al.^20^ observed dose-dependent increases in BWG of broiler chickens with rising FR levels. However, some studies, such as Mahmoudi et al.^21^, did not report significant effects in quail, suggesting species- or dosage-dependent responses. Regarding MO, our findings support the work of Kwiecień et al.^22^, Kasapidou et al.^23^, who reported increased BW and BWG in broilers fed MO -supplemented diets. The growth-promoting effects are likely due to the plant’s flavonoid and polyphenol content, which exert antioxidant, antimicrobial, and appetite-stimulating activities. Others, like Poorghasemi et al.^12^, Skomorucha and SosnówkarCzajka^24^, did not find significant MO effects, suggesting that effectiveness may depend on concentration, duration, or form of administration. Crucially, the observed improvements in BW and BWG occurred without a significant increase in total FI across any treatment groups. This agrees with the results of Amer et al.^25^, Guerrini et al.^26^ who also found that FR supplementation did not affect feed consumption. Similarly, Poorghasemi et al.^12^ reported that lemon balm extract had no impact on FI. The lack of change in FI alongside improved BWG and FCR suggests that FR and MO improved feed efficiency rather than intake quantity. This notion is strongly supported by the significant improvement in FCR among quails receiving 10 g FR, 12 g FR, and 3 ml MO, indicating more efficient nutrient utilization. These findings mirror those of Mohamed et al.^20^, Amer et al.^25^, who observed dose-dependent improvements in FCR with FR supplementation. Similarly, the improved FCR in the MO group aligns with findings by^23,27^. The white quail strain outperformed the brown strain in BW, BWG, and FCR, consistent with Kamel et al.^28^, who attributed superior growth performance to the genetic potential of the white genotype.

Regarding carcass traits, significant interactions (*P* < 0.05) between T and S for dressing percentage, liver, and gizzard weight are in agreement with Kamel et al.^28^, who observed significant strain × diet interactions for carcass yield in quails fed herbal blends. Considering treatment effects, 10 g FR significantly increased dressing percentage, reaching 74.61%, compared to 73.05% in the control group. These findings align with Al-Yasiry et al.^9^, who observed a linear increase in total carcass muscle and a reduction in abdominal fat content with increasing Boswellia serrata resin levels. However, our findings disagree with those reported by Ismail et al.^18^, Al-Yasiry et al.^29^, who found no significant effect of Boswellia serrata resin or FR supplementation on dressing percentage in broilers and rabbits, respectively. In terms of internal organs, 12 g FR supplementation resulted in a significant increase in liver percentage (3.26%), which supports Ismail et al.^18^, Mohamed et al.^20^, who observed significant changes in organ weights, including liver hypertrophy, upon phytogenic supplementation. The observed increase in liver weight in our study likely reflects enhanced metabolic and anabolic activity rather than pathological enlargement^30^. This interpretation is supported by the upregulated expression of *IGF1* and *GPX1*, indicating stimulated growth processes and improved antioxidant capacity, as discussed further in the gene expression section. However, our findings disagree with Mohamed et al.^20^ in terms of carcass yield, as they reported no significant effect of Boswellia serrata resin on carcass or dressing percentages in chicks. Supplementation with MO also showed notable effects on carcass traits. Birds receiving 3 ml MO had significantly higher dressing percentages (73.89%) and increased spleen and gizzard weights compared to controls. These results are consistent with Kwiecień et al.^22^, Kasapidou et al.^23^, who documented enhanced carcass yield in broilers fed diets enriched with MO at different levels. The improvement in gizzard weight (especially in the 2.5 ml MO group) and spleen weight (0.14% in both 2.5- and 3-ml MO groups) highlights the potential of MO to promote digestive and immune organ development. However, our findings contradict several studies that found no effect of MO on carcass yield or internal organ weights, including^12,31,32^. These discrepancies may be attributed to differences in the method of administration (feed vs. water), dosage levels, or the species studied, as our research was conducted in Japanese quail, which may respond more sensitively to phytogenic than broilers. Strain differences were significant for dressing percentage, liver, and gizzard weights (*P* < 0.05), with white quails showing higher dressing (74.02%) and gizzard weight (2.83%), while brown quails had heavier livers (3.14%). These results partially align with Kamel et al.^28^, Kamel et al.^17^, Kirrella et al.^33^, who reported that white-feathered quails had superior carcass weights compared to brown ones. Similarly, Nasr et al.^34^ reported that white quails had the highest values of dressing percentage and internal organ weights compared to brown and golden strains, supporting our findings. However, Shehata et al.^35^, Sabow^36^ reported no significant differences in carcass traits between strains, highlighting that strain responses may vary based on environment, nutrition, and physiological status.

The sensory evaluation results revealed that no significant T × S interaction was detected for any sensory attribute, indicating that the response to dietary supplementation was similar across both quail strains. This suggests that the effects of PFAs on meat sensory quality are not dependent on genetic background. At the treatment level, dietary supplementation with FR and MO significantly improved most meat quality attribute with no adverse effects on taste. These findings indicate that the use of PFAs not only enhanced growth traits but also contributed positively to the sensory properties of quail meat. Lipid oxidation is a major factor influencing meat quality deterioration, particularly in terms of flavor, color, texture, and nutritional value^37,38^. This process, triggered by the reaction of polyunsaturated fatty acids with reactive oxygen species, leads to oxidative rancidity, off-flavors, and discoloration. Therefore, the inclusion of natural antioxidants in poultry diets is critical to minimizing oxidative stress and maintaining meat quality, particularly under intensive commercial production conditions^39^. Regarding FR, although limited studies have focused on its sensory effects, Kiczorowska et al.^40^ showed that dietary inclusion of Boswellia serrata resin enhanced the physicochemical properties of breast and drumstick muscles, improving water-holding capacity and reducing cooking losses, which likely translates into better tenderness and juiciness. The improvement in aroma and tenderness observed in all additive groups in our study may be attributed to such physicochemical enhancements, possibly mediated by boswellic acids and other active terpenes found in FR. MO, known for its high antioxidant potential due to compounds such as rosmarinic acid and flavonoids, is one such promising additive. Our findings are supported by Marcinčák et al.^41^, who demonstrated that supplementation with lemon balm improved the taste, juiciness, and tenderness of chicken meat, particularly after long-term frozen storage. Additionally, studies by Kasapidou et al.^23^, Eleroğlu et al.^42^ reported that MO supplementation contributed to a lighter breast muscle color in broilers a trend consistent with our observation of significantly improved color scores in the MO-treated groups. These findings align closely with Elkhoriby et al.^43^, who reported that supplementation with a frankincense–melissa mixture in drinking water markedly improved the sensory quality of meat in both black and white Japanese quail strains, irrespective of their genetic background. At the strain level, no significant differences were observed between brown and white quails for any sensory parameter, confirming that sensory responses were consistent across genotypes.

The economic analysis revealed that the significant interaction between T and S highlights that economic response is not uniform across genotypes. In the brown strain, the control and 10 g FR groups yielded the best economic outcomes, whereas in the white strain, the 10 g FR and 3 ml MO groups were economically superior. Interestingly, the 3 ml MO group achieved the highest EE% in white quails, indicating that MO supplementation may be more economically efficient in genetically higher performing strains. This interaction aligns with results from Kamel et al.^17^ on the significance of the genetic type of quail and dietary treatment interactions in economic evaluation parameters. Taken together, these findings suggest that while high doses of FR increase production costs, moderate levels (10 g FR) and MO supplementation (3 ml/L) can strike a balance between biological efficacy and economic gain. Given the scarcity of literature on the economic evaluation of FR, the present study contributes novel insights and serves as a foundation for further investigation into the cost effectiveness of phytogenic additives in quail production. Across treatments, dietary supplementation with FR and MO significantly influenced production cost and economic return indicators. The 12 g FR group showed the highest TVC and total cost (TC), reflecting the additional expense associated with higher supplementation levels. However, this group also recorded a significantly lower net return (NR) and gross margin (GM), suggesting that the cost increment was not matched by proportional gains in productivity or market value. Conversely, the 10 g FR group achieved the most favorable economic outcomes, including significantly higher net return and gross margin, and the highest relative gross margin (RGM) along with the 3 ml MO group. These results suggest that moderate levels of FR and MO optimize both performance and economic profitability. To date, limited research has evaluated the economic impact of FR supplementation in poultry. However, Al-Yasiry and Kiczorowska^19^ reported that broilers receiving 3 g Boswellia serrata resin/L in drinking water exhibited the highest economic efficiency index, supporting our finding that intermediate dosages (such as 10 g/L) are more cost-effective than higher ones. The reduced economic efficiency observed in the 12 g FR group in our study further supports this concept. To the best of our knowledge, no published studies have evaluated the economic impact of MO supplementation in poultry. However, the favorable economic returns and efficiency metrics observed in the 3 ml MO group, particularly in the white quail strain, may be attributed to the plant’s growth-promoting and antioxidant effects, which enhanced production outcomes without incurring high input costs. These results highlight the potential of MO as a cost-effective natural additive and underscore the need for future economic assessments across different production systems. Regarding strain effects, the white strain exhibited significantly higher costs, returns, and net return than the brown strain. This is likely a reflection of the higher BW and FI, which resulted in increased input costs but also enhanced revenue from final BW and market value. Moreover, the white strain demonstrated better economic efficiency (EE%) and gross margin, emphasizing its superior economic potential in quail meat production. In the same line, Kamel et al.^17^ found that the selling return of white quails was significantly higher than that of the brown strain. Similarly, Kamel et al.^28^ reported that the white strain also had the best TR, surpassing the brown strain. However, Mustafa and Sulaiman^44^ found that no significant differences were noted among the brown, black, and white lines in economic profit.

Regarding gene expression, to the best of our knowledge, this is the first study to examine the effects of dietary supplementation with FR and MO on the expression of growth-, antioxidant-, and immunity-related genes in Japanese quail. The present study demonstrated a significant T × S interaction in the expression of growth-, antioxidant-, and immunity-related genes in Japanese quail, as shown in Fig. 1. These findings emphasize that the molecular response to PFAs is not only treatment-dependent but also strongly influenced by genetic background. In brown quails, supplementation with 10 and 12 g/L FR resulted in the most pronounced improvements in growth and oxidative balance, as reflected by upregulated *IGF-I*, suppressed *MSTN*, and elevated *GPX1* expression. Conversely, the 3 mL/L MO and 10 g/L FR treatments consistently enhanced *IGF-I* expression, *MSTN* downregulation, *GPX1* activity, and *IL-6* expression, suggesting a synergistic effect across growth, immunity, and antioxidant pathways.

At the treatment level, the current findings demonstrate that PFAs exert distinct modulatory roles depending on inclusion level and gene target. Supplementation with 3 mL/L MO and 10 g/L FR produced the most favorable growth-related responses, characterized by upregulated *IGF-I* and suppressed *MSTN* expression followed by 12 g/L FR compared with the control and 2.5 mL MO. *IGF-I* is a key anabolic regulator involved in somatic growth, protein synthesis, and feed efficiency^28,45,46^. The observed upregulation of *IGF-I* by MO and FR supplementation is consistent with earlier reports where other phytogenic or natural additives, such as thymol, pomegranate peel, and Paulownia leaf extract, enhanced hepatic *IGF-I* expression alongside improvements in growth performance. In contrast, the downregulation of *MSTN*, a well-known negative regulator of skeletal muscle development, was most evident in the 10 g FR group, suggesting enhanced muscle accretion potential^47,48^.

The marked differences in gene expression observed between relatively close supplementation levels (10 vs. 12 g/L FR and 2.5 vs. 3 mL/L MO) may reflect non-linear dose–response relationships typical of phytogenic bioactive compounds^49,50^. Unlike conventional nutrients, phytogenic additives function as signaling modulators that influence transcriptional regulation through antioxidant, endocrine, and immune pathways^51,52^. Bioactive constituents such as boswellic acids in frankincense and polyphenols and flavonoids in MO can activate intracellular signaling cascades once a physiological threshold concentration is achieved^53,54^. Crossing this threshold may trigger amplified activation of growth-related pathways, particularly the IGF-1 axis, alongside suppression of negative regulators such as MSTN^55^. Consequently, even small increases in supplementation level may induce disproportionately large transcriptional responses, a phenomenon widely described in nutrigenomic regulation of livestock species^56^.

Previous poultry studies have similarly shown that nutritional interventions such as methionine supplementation^57^ or high-protein/energy diets^58^ downregulate *MSTN*, thereby improving growth and carcass yield. Taken together, these results suggest that bioactive compounds in FR and MO may influence growth performance at least partly through molecular regulation of the *IGF-I* and *MSTN*. Dietary treatments also altered hepatic *GPX1* expression, with the 10 and 12 g/L FR and 3 ml MO groups showing the highest values. This indicates a strong antioxidant-promoting role for FR and MO particularly at higher concentrations. GPX1 is a crucial antioxidant enzyme that detoxifies hydrogen peroxide and lipid hydroperoxides, thereby protecting tissues against oxidative damage^59,60^. Our findings align with previous evidence that dietary bioactive compounds and trace minerals can upregulate *GPX1* expression. For example, selenium-enriched diets in broiler breeders^61^ and zinc oxide nanoparticle supplementation in quail^62^ both elevated hepatic *GPX1* levels. The current results therefore highlight the potential of FR, especially at higher inclusion levels, to strengthen the antioxidant defense system in quail. The most pronounced immune response was observed in birds receiving 3 mL/L MO which showed the highest *IL-6* expression across treatments. *IL-6* is a multifunctional cytokine central to innate and adaptive immunity, acute-phase responses, and hematopoiesis^63^. Its upregulation by MO may be attributed to bioactive components such as flavonoids and polyphenols, which are known to modulate immune signaling pathways^64^. Comparable effects have been reported in poultry supplemented with β-glucans and ascorbic acid, which significantly altered *IL-6* transcription in chicken spleens^65,66^. Although direct evidence on MO or FR is lacking, the present findings support the immunostimulatory role of herbal additives in quail, potentially enhancing resistance to pathogens and stress.

As shown in Fig. 3, the relative expression of the studied genes differed between brown and white quail strains. The white quail strain showed higher expression of *IGF-1* and lower *MSTN* levels than the brown strain, indicating superior growth potential. Conversely, the brown strain expressed higher levels of *GPX1* and *IL-6*, reflecting stronger antioxidant and immune gene activity. The higher *IGF-I* expression in white quails supports their enhanced growth potential, consistent with the findings of Gasparino et al.^67^, who demonstrated that high-feed-efficiency quails exhibited elevated hepatic *IGF-I* expression, particularly under stress conditions. Similarly, these results align with Hosnedlova et al.^68^, who reported that fast-growing chickens displayed higher hepatic *IGF-I* mRNA expression and circulatory *IGF-I* concentrations compared to slower-growing counterparts. On the other hand, the brown strain exhibited higher expression levels of *GPX1* and *IL-6*, this indicates that the brown strain may be genetically predisposed to stronger antioxidant responses, which could be advantageous under environmental or nutritional stress. This observation agrees with Shehata et al.^35^, who reported greater *IL-6* expression in pigmented quails compared with white ones when fed mulberry leaf–supplemented diets.

**Fig. 4:**
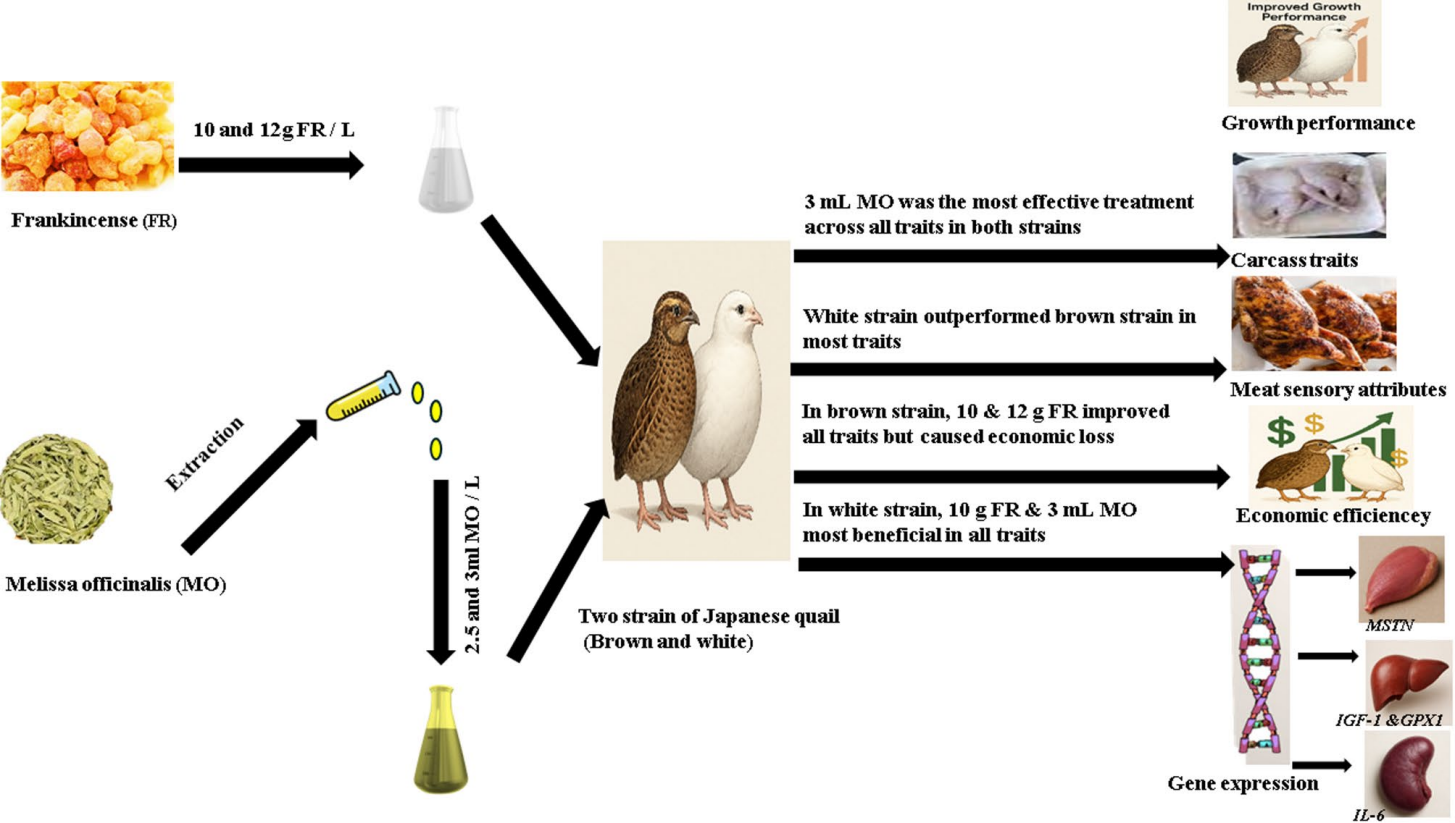
Schematic representation of the effects of frankincense and *Melissa officinalis* supplementation on performance, carcass traits, meat quality, economic efficiency, and gene expression in two strains of Japanese quail.

In conclusion, the present study demonstrates that the interaction between treatment and strain played a critical role in shaping the productive and economic responses of Japanese quail. The results clearly indicated a strain-dependent response to phytogenic supplementation, where the white strain exhibited the most favorable overall performance when supplemented with 3 mL/L MO, achieving improvements in growth performance, feed efficiency, carcass traits, meat quality, economic returns, and the expression of key genes related to growth (*IGF-1*↑, *MSTN*↓), antioxidant status (*GPX1*↑), and immunity (*IL-6*↑). In contrast, although the brown strain showed a stronger biological response to higher FR levels (12 g/L) in terms of growth and gene expression, this response was not economically efficient, highlighting the importance of considering both biological and economic outcomes (Fig. 4). Regarding the treatment effects, supplementation with MO, particularly at 3 mL/L, and FR at 10 g/L consistently improved most evaluated traits compared to the control. The white strain generally outperformed the brown strain in productive efficiency and economic return. The findings also support the broader adoption of phytogenic feed additives as sustainable strategies to improve poultry performance, product quality, and economic profitability while reducing reliance on synthetic growth-promoting agents.

## Materials and methods

### Birds, housing, and management

A total of 300 fourteen-day-old female Japanese quail chicks of two strains, brown (*n* = 150) and white (*n* = 150), were procured from a commercial farm in Kafr El-Sheikh, Egypt. The initial body weights (BW) were 57.44 ± 0.02 g for the brown strain and 57.44 ± 0.04 g for the white strain. The birds were housed in wire battery cages (150 × 50 × 30 cm) within a conventional, ventilated poultry house. A layer of sawdust covered with corrugated paper was used as litter. Each cage was equipped with a feeder and a drinker, providing ad libitum access to feed and water. The ambient temperature was maintained at approximately 29 °C. Standard hygienic protocols were strictly followed throughout the experiment. All protocols were carried out in accordance with guidelines and regulations of the Universal Directive on the Protection of Animals Used for Scientific Purposes. Birds were observed daily by trained personnel for clinical signs of illness, discomfort, or distress. Humane endpoints were established in advance and included inability to stand or ambulate, persistent or severe distress, unresponsiveness to external stimuli, serious injury, or a loss exceeding 20% of body weight. Animals exhibiting any of these criteria were promptly euthanized by decapitation in accordance with the American Veterinary Medical Association (AVMA) Guidelines for the Euthanasia of Animals (2020) to minimize pain and prevent further suffering. All protocols follow the ARRIVE guidelines for reporting animal research (https://arriveguidelines.org).

### Experimental design, diets, and preparation of PFAs

All birds received a basal diet formulated to meet or exceed the nutritional requirements recommended by the National Research Council (NRC, 1994), as detailed in Table 9. For each quail strain, birds were randomly allocated into five treatment groups (30 birds per treatment), with each treatment consisting of three replicates of 10 birds each.

**Table 9:** Composition and calculated analysis of the basic quail diet fed during the experimental period.

Feed ingredients	Growing diet (Kg)
Yellow corn	52.2
Soyabean meal (44%CP)	39.1
Corn gluten	4
Mixed oil	0.575
Limestone	1.75
Di-calcium phosphate	1.3
Salt	0.3
Vit and min premix^1^	0.3
Lysine	0.18
DL- methionine	0.16
Energy enzyme^2^	0.025
Phytase enzyme^3^	0.01
Antitoxin	0.1
Total	100
Calculated analysis	
ME (Kcal / Kg)	2900
Crude protein	24
Ether extract	02.89
Crude fiber	03.61
Calcium %	0.912
Total phosphorus	0.691
Available phosphorus	0.447
Lysine %	1.345
Methionine	0.586

Vitamin and mineral premix^1^(Vita Care): Each 3 kg of the premix contains the following: vitamin A = 2,500,000 IU, vitamin D3 = 2,500,000 IU, vitamin E = 5,000 mg, zinc sulfate = 50 g, copper sulfate = 15 g, ferric sulfate = 30 g, cobalt sulfate = 200 mg, potassium iodide = 600 mg, sodium selenite = 220 mg, magnesium sulfate = 20 g, manganese sulfate = 50 g.

Energy enzyme supplement^2^(Natozyme Mix): Each 1 g of the supplement contains the following: xylanase = 15,000 U, ß-mannanase = 2,000 U, α-galactosidase = 500 U, α-amylase = 800 U, acid protease = 6,000 U, neutral protease = 6,000 U, cellulase = 1,000 U, glucose oxidase = 1,500 U, pectinase = 500 U. The carrier is corn starch, which is added up to 1 g.

Phytase enzyme supplement^3^ (PHYTASE_5000): Each 1 g of the supplement contains phytase (from E. coli) at a dosage of 5,000 units. The carrier is calcium carbonate, which is added up to 1 g.

FR resin and dried MO leaves were purchased from a commercial supplier. The FR aqueous extract was prepared by mixing 10–12 g of fragmented resin per liter of drinking water and allowing it to dissolve for 12 h before administration, following the method described by Al-Yasiry and Kiczorowska^19^. The MO extract was prepared by infusing 200 g of dried leaves in 1 L of boiling water for 10 min. The infusion was then cooled to 40 °C and strained^12^. Therefore, both phytogenic additives were administered to the birds through drinking water as aqueous extracts, ensuring a consistent delivery method across treatments. Phytochemical screening of both FR and MO was conducted via High-Performance Liquid Chromatography (HPLC) at the Faculty of Pharmacy, Mansoura University, to characterize their respective major active constituents. The analytical procedure for frankincense was conducted in line with the settings described by Asteggiano et al.^69^, while the method for MO was adapted from Arceusz and Wesołowski^70^. By comparing retention times and UV spectra with their corresponding reference standards, the analysis successfully quantified the primary biomarkers in both plants. In the FR, 3-O-acetyl-11-keto-β-boswellic acid was identified as the predominant triterpene (4.87%), followed by β-boswellic acid (2.21%), 11-keto-β-boswellic acid (1.53%), and α-boswellic acid (1.04%). Conversely, the analysis of the MO extract revealed rosmarinic acid as the major phenolic compound (4.15%), along with minor quantities of chlorogenic acid (0.82%), caffeic acid (0.45%), and luteolin (0.28%).

The inclusion levels used in the present experiment were selected based on relevant literature Al-Salihy and Al-Hussaini^71^ and a preliminary trial conducted prior to the main study to identify appropriate supplementation levels for Japanese quail.

### Data collection

Growth performance parameters were recorded weekly throughout the experimental period. Chicks were initially weighed in groups, with each replicate weighed individually to the nearest gram using a digital scale with a digital display (SF400, 10 kg capacity) with an accuracy of ± 1 g.

Body weight gain (BWG) was calculated weekly following the method described by Mashayekhi et al.^72^, by subtracting the previous week’s BW from the current week’s value. Feed intake (FI) was calculated by subtracting the leftover feed from the total amount offered^73^. Feed conversion ratio (FCR) was then determined using the formula FCR = FI / BWG^74^.

At 42 days of age, carcass traits were assessed by randomly selecting three birds per replicate (nine birds per treatment group) to avoid selection bias and ensure a representative sample of each replicate. The selected birds were fasted for 12 h and then weighed before the procedure. Birds were humanely euthanized by decapitation using a sharp blade by a trained veterinarian on-site at the Poultry Research Unit of the Faculty of Veterinary Medicine, Mansoura University. No prior anesthesia was administered, and the method followed the AVMA Guidelines for the Euthanasia of Animals (2020). Death was confirmed by the absence of corneal reflexes and heartbeat before tissue collection.

Post-slaughter, the hot carcass weight and the weights of the liver, heart, gizzard, and spleen were recorded. Dressing percentage was calculated as the hot carcass weight divided by live BW and multiplied by 100^75^. The relative organ weights were calculated as a percentage of live BW, as described by Zhu et al.^76^, using a digital balance (MH-Series pocket scale, 200 g capacity, 0.01 g accuracy).

Sensory evaluation of breast meat was performed using samples from three birds per treatment group. The meat was cooked without spices (only salt added) and evaluated by a trained panel of 12 members from the Department of Food Safety, Faculty of Veterinary Medicine, Mansoura University. The panel assessed color, aroma, taste, tenderness, juiciness, and overall acceptability using a 9-point hedonic scale (1 = dislike extremely, 9 = like extremely) (Table 10), following the method of Ruiz-Capillas et al.^77^.

**Table 10:** Hedonic scale score card for the evaluation of quail meat.

Attributes	Score	
Color Aroma Juiciness Tenderness Taste Overall acceptability	9	Like extremely
	8	Like very much
	7	Like moderately
	6	Like slightly
	5	Neither like nor dislike
	4	Dislike slightly
	3	Dislike moderately
	2	Dislike very much
	1	Dislike extremely

For economic analysis, total variable costs (TVC) were calculated based on feed, chicks, labor, veterinary services, and other production-related inputs, as outlined by Bano et al.^78^. Total fixed costs (TFC) included depreciation of land, buildings, and equipment, calculated using a 25-year depreciation for buildings and 5 years for equipment^79^. Total costs (TC) were derived by summing TVC and TFC^80^. Total return (TR) was determined based on the market BW of quails at six weeks and the value of litter^17^. Net return (NR) was calculated by subtracting TC from TR^81^. Gross margin (GM) was the difference between TR and TVC as described by Emokaro and Eweka^82^, while the relative gross margin (RGM) was calculated as the GM of each treatment relative to that of the control group^83^. Economic efficiency was evaluated by dividing NR by total feed costs (including additives) and multiplying by 100^84^.

Regarding gene expression analysis, 30 birds (three per treatment per strain) were slaughtered at six weeks of age, and liver, muscle, and spleen tissues were collected and preserved in RNAlater^®^ solution at − 80 °C to maintain RNA integrity^85^. Total RNA was isolated using the miRNeasy Mini Kit (Qiagen, Cat. No. 217004). Complementary DNA (cDNA) was synthesized using the High-Capacity cDNA Reverse Transcription Kit (Thermo Fisher Scientific, Cat. No. 4368814). Quantitative real-time PCR (qRT-PCR) was carried out using Maxima SYBR Green qPCR Master Mix (2X) (Thermo Scientific, Cat. No. K0251). The PCR master mix for each 25 µl reaction consisted of 12.5 µl SYBR Green Master Mix, 0.3 µl each of forward and reverse primers, 10 nM ROX solution, 500 ng cDNA, and nuclease-free water. Gene expression levels of *IGF-I*, *MSTN*, *IL-6*, and *GPX1* were quantified using gene-specific primers (Table 11), and *β-actin* was used as the endogenous housekeeping gene. The final reaction mixture was placed in a thermal cycler, and the following program was carried out: reverse transcription at 50 °C for 30 min, primary denaturation at 95 °C for 10 min followed by 40 cycles of 94 °C for 15 s, annealing temperature at 55 °C for 1 min, and 72 °C for 30 s. The 2^−ΔΔCt method was used to determine the relative mRNA expression levels, normalized to *β-actin*^86^.

**Table 11:** Forward and reverse primer sequences and accession numbers of *IGF-I*, *MSTN*, *IL-6*, and *GPX1* genes.

Gene	Source of isolation	Primer sequence	Accession number	Reference
*IGF-I*	Liver	F: 5′- CACCTAAATCTGCACGCT − 3′ R: 5′- CTTGTGGATGGCATGATCT − 3′	AF260131.1	^15^
*MSTN*	Muscle	F:5′-GGTATCTGGCAGAGTATTGATGTGAA3′ R: 5′-CAAAATCTCTGCGGGACCGT − 3′	XM_015867858.2	^88^
*IL-6*	Spleen	F: 5′- CAACCTCAACCTGCCCAA − 3′ R: 5′- GGAGAGCTTCCTCAGGCATT − 3′	AB559572.1	^14^
*GPX1*	Liver	F: 5′- CAGTTCGGGCATCAGGAGAA-3′ R:5′CGAGGAACTTGCTCGAAAGTTACCAGG-3′	AB371709.1	^16^
*β-actin*	–	F: 5′- CTGGCACCTAGCACAATGAA-3′ R: 5′- CTGCTTGCTGATCCACATCT − 3′	AF199488	^14^

### Statistical analysis

Data was analyzed using the General Linear Model (GLM) procedure of SPSS (Version 21). The statistical model included the fixed effects of treatment, quail strain, and their interaction.

$${{\mathrm{Y}}_{{\mathrm{ijk}}}}\,=\,{{\mu}}\,+\,{{\mathrm{T}}_{\mathrm{i}}}+{{\mathrm{S}}_{\mathrm{j}}}+{\left( {{\mathrm{T}} \times {\mathrm{S}}} \right)_{{\mathrm{ij}}}}+{{\mathrm{e}}_{{\mathrm{ijk}}}}$$

Where: Y_ijk_ is the observed value, µ is the overall mean, T_i_ is the effect of treatment, S_j_ is the effect of the j strain, (T×S)_ij_ is the interaction effect, and e_ijk_ is the random error.

Mean comparisons among treatments were re-analyzed using the Tukey–Kramer multiple comparison test^87^. The results are presented as least squares mean ± standard error, which provides a measure of the variability in the data.

The normality of sensory score data obtained using hedonic scales (1–9) was examined using the Shapiro–Wilk test. The results confirmed that the sensory traits across the treatment groups were normally distributed and ranged from (0.07 to 1).

## Data Availability

The datasets used and/or analyzed during the current study are available from the corresponding author upon reasonable request.
